# Observation of pressure induced charge density wave order and eightfold structure in bulk VSe_2_

**DOI:** 10.1038/s41598-021-97630-8

**Published:** 2021-09-13

**Authors:** Zhiying Guo, Xingyu Hao, Juncai Dong, Haijing Li, Jiangwen Liao, Dongliang Chen

**Affiliations:** 1 Beijing Synchrotron Radiation Facility, Institute of High Energy Physics, Chinese Academy of Sciences, Beijing, 100049 China; 2 University of Chinese Academy of Sciences, Beijing, 100042 China

**Keywords:** Condensed-matter physics, Ferromagnetism, Structure of solids and liquids

## Abstract

Pressure-induced charge density wave (CDW) state can overcome the low-temperature limitation for practical application, thus seeking its traces in experiments is of great importance. Herein, we provide spectroscopic evidence for the emergence of room temperature CDW order in the narrow pressure range of 10–15 GPa in bulk VSe_2_. Moreover, we discovered an 8-coordination structure of VSe_2_ with *C2/m* symmetry in the pressure range of 35–65 GPa by combining the X-ray absorption spectroscopy, X-ray diffraction experiments, and the first-principles calculations. These findings are beneficial for furthering our understanding of the charge modulated structure and its behavior under high pressure.

## Introduction

Layered 1*T*-VSe_2_, a typical transition metal dichalcogenide (TMDCs), is an excellent candidate material for the next-generation electronic application and tunable optoelectronic device^1–5^. It owns many novel physical properties and exhibits a rich variety of correlated electronic phenomena, such as charge-density-wave (CDW) state^6^, high-pressure superconducting^7^, photoinduced insulator–metal transition^8^, and possible ferromagnetic order in its monolayer form^9–11^. Among them, the CDW is a low-temperature condensed phase that is featured by periodic modulation of charge densities accompanied with spontaneous lattice distortion^12^. It receives much scientific and technological attention and has realized in many metallic layered TMDCs, such as VX_2_, NbX_2_, and TaX_2_ (where X = Se, Te, etc.)^13–18^. In order to manipulate the CDW order, understanding how it evolves with pressure or substrate stress is of paramount importance.

**Figure 1:**
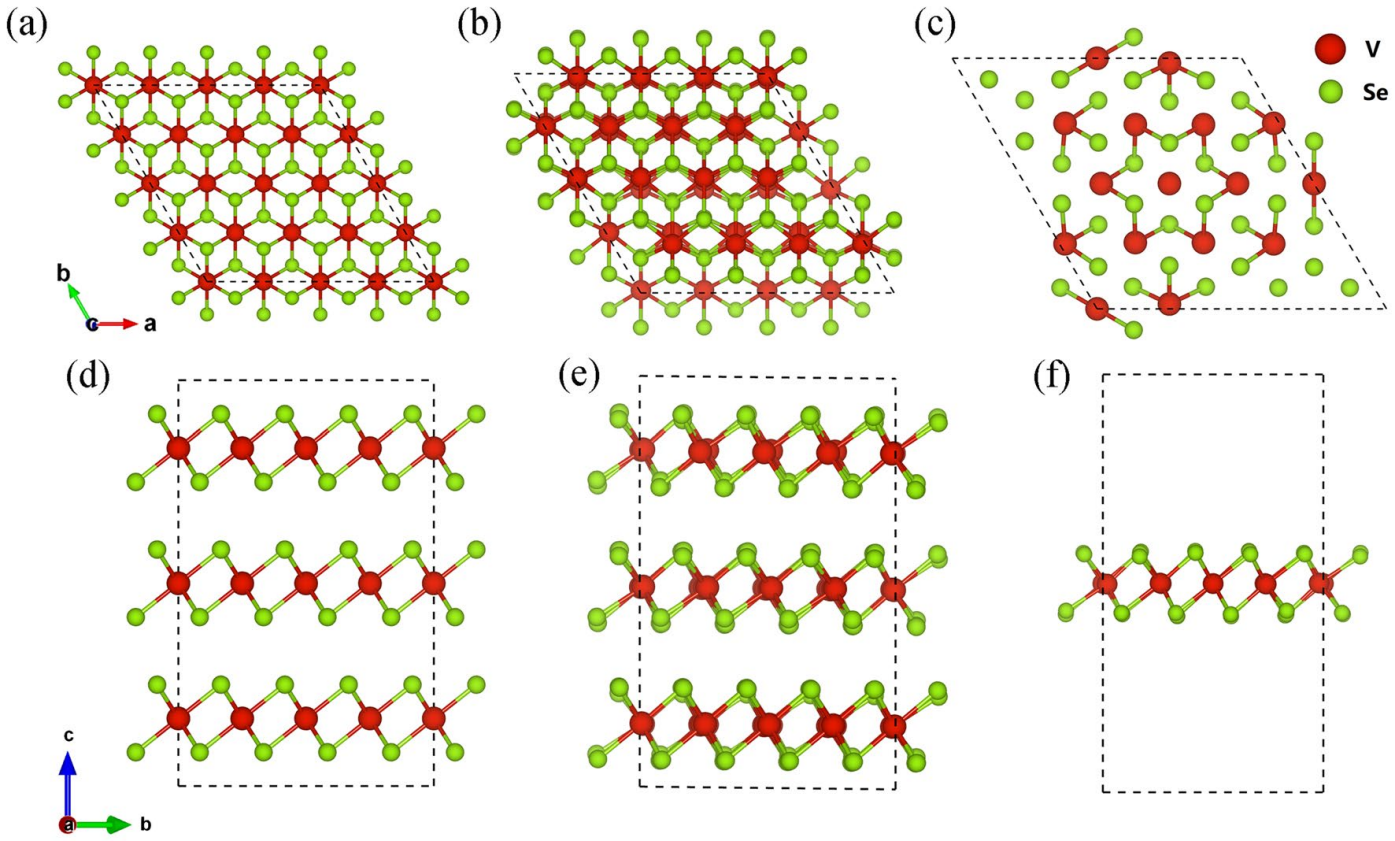
Top view and side view of the crystal structure of bulk 1T-VSe_2_ and its 3D/2D CDW orders. Top view (**a**) and side view (**d**) of 4 × 4 × 3 supercell of normal state 1T-VSe_2_. Top view (**b**) and side view (**e**) of 4a × 4a × 3c superstructure of 3D-CDW state (having commensurate in-plane wave vector with an incommensurate out-of-plane component). Top view (**c**) and side view (**f**) of 4 × 4 2D-CDW state of VSe_2_ monolayer.

VSe_2_ crystallizes in the 1*T* polytype with a space group of $P\overline{3}m1{ }$(CdI_2_-type structure) at ambient conditions, where the V atoms are covalently bonded with the octahedra of Se atoms to form a Se-V-Se layer and then these layers are linked through weak van der Waals forces as shown in Fig. 1a,d. 1*T*-VSe_2_ undergoes an incommensurate CDW transition around 110 K and commensurate CDW transition around 80 *K*^19^ driven by the conventional Fermi surface nesting mechanism^20^ or the newly proposed electron–phonon coupling^21^, forming a 4a × 4a × 3c superstructure as shown in Fig. 1b,e. Room-temperature CDW order in bulk VSe_2_ has recently been observed by Raman spectroscopy under hydrostatic pressure of 7.4–14.9 GPa^22^. Pressure-induced *T*_CDW_ enhancement can overcome the limitation of low temperature and offers a promising route for the development of CDW based electronic devices. Experimental determination of the lattice distortion and structural differences between the normal state and the CDW state is always the priority for any CDW study. Because the distortion or the atomic displacements in CDW state is extremely small (e.g., 0.1 ~ 0.15 Å in VSe_2_), the superstructure would only produce relatively weak satellite peaks in the X-ray diffraction pattern^23,24^. Thus, seeking the traces of pressure-induced CDW by X-ray diffraction or absorption spectroscopy techniques is challenging but highly desired in practice.

On the other hand, reduced dimensionality and interlayer coupling in van der Waals materials gives rise to fundamentally different electronic, magnetism, and multiple charge density orders in monolayers compared with the bulk^9,25^. Due to the different preparation conditions, especially the substrate and strain conditions, there are contradictory reports about the magnetism of the VSe_2_ monolayer. Strong ferromagnetism up to room temperature was reported in monolayer VSe_2_ on highly oriented pyrolytic graphite and MoS_2_ substrates^9^. While some other groups found multiple CDW orders with paramagnetic properties. For instance, Chen et al*.* found a $\sqrt 7 \times \sqrt 3$ CDW superstructure in 1*T*-VSe_2_ monolayer/bilayer graphene^26^. Feng et al*.* discovered an enhanced $4 \times 4$ CDW order with the temperature near 140*K*^27^, in which the so-called Star of David (SoD) unit^28^ can be formed in the center as shown in Fig. 1c,f. In addition, 2 $\times \;\sqrt 3$ and 4 $\times \;\sqrt 3$ CDW orders with a transition temperature of 350 and 100 K have also been reported^29,30^. A combined study of scanning tunneling microscopy and angle-resolved photoemission spectroscopy clearly demonstrates that the multiple CDW phases in monolayer VSe_2_, as well as its topography structures, are sensitive to different graphene substrates and interlayer couplings^31,32^. The ferromagnetism order is suppressed in those multiple CDW orders, where the underlying substrate or interface may play important roles such as charge transfer or strain^33^.

At last, tuning the physical properties of the material by applying pressure or by strain requires an understanding of its ground-state crystal structure. 1*T*-VSe_2_ is stable under ambient condition^34^. Researchers have achieved great advances in the chemical vapor transport growth of high-quality and large-size 1T-VSe_2_ single crystals^35^. Under high pressure, previous studies mainly focus on the pressure range of 0–30 GPa, and a first-order phase transition has been reported to occur at 15 GPa^36^. The second phase of VSe_2_ possess attractive superconducting properties^7^, and has been assigned to be the monoclinic NbTe_2_-type 1*T′* structure (space group *C2/m*), which are labeled as *C2/m*-I here in order to distinguish it from another *C2/m* structure. All reported VSe_2_ structures consist of six-coordinated V atom. The ground state structure of VSe_2_ in a higher-pressure range (e.g. 30–65 GPa) or with a higher V coordination number has not been reported yet.

In this work, from the above-mentioned aspects, we systematically studied the bulk VSe_2_ system using high-pressure X-ray diffraction (XRD), X-ray absorption fine structure (XAFS) spectroscopy, combining with the first-principles calculations. Herein, we reported two experimental traces of the pressured-induced CDW transition in bulk VSe_2_. In addition, we identified an 8 coordination *C2/m*-II structure and established a phase transition pathway of $1{\text{T}}\mathop{\longrightarrow}\limits^{{15\;{\text{GPa}}}}$ *C2/m*-${\text{I}}\mathop{\longrightarrow}\limits^{{35\;{\text{GPa}}}}$ *C2/m*-II (8 coordination) in the pressure range of 0–65 GPa.

## Methods

### High-pressure XRD measurements

Bulk 1T-VSe_2_ crystals were commercially purchased from 2D semiconductors Inc. and Nanjing 2DNANO Tech. Co., Ltd. In situ high-pressure XRD experiments were performed at the 4W2 beamline of the Beijing Synchrotron Radiation Facility (BSRF) by angle-dispersive measurements with a wavelength of 0.6199 Å and focused x-ray beam size of 26 × 8 μm^2^ (FWHM). Pressure was generated by a symmetric piston-cylinder type diamond anvil cell (DAC) with a pair of diamond anvils with a culet size of 300 μm. A rhenium (Re) gasket pre-indented to 45 μm in thickness with a drilled hole of 120 μm in diameter was used as the sample chamber. The VSe_2_ crystals were ground into polycrystalline powder, and then loaded into the chamber with methanol-ethanol–water (16:3:1) mixture as the pressure-transmitting medium (PTM), which provides quasi-hydrostatic condition up to 11 GPa^37^ and has been used in high-pressure XRD experiments for layered materials such as VSe_2_, TaS_2_, and BiSe_2_. No evidence of interaction between VSe_2_ and methanol: ethanol: water was reported or observed. Two rounds of experiments were performed. Pressure was determined by the ruby fluorescence technique^38^. The diffraction patterns were collected by a PILATUS detector and integrated using the FIT2D software^39^, and Rietveld refinements on high-pressure data were completed by the GSAS-II package^40^.

### High-pressure XAFS measurements

The Se *K*-edge XAFS spectra of VSe_2_ were measured in the transmission mode at 1W2B beamline of BSRF by a combination of single crystal DAC and polycapillary half-lens to suppress the DAC glitches^41^. A rhenium (Re) gasket pre-indented to 60 μm thick with a drilled hole of 120 μm in diameter was used as the sample chamber. Bulk 1*T*-VSe_2_ crystals were finely grounded and homogeneously mixed with LiF as pressure-transmitting medium and then loaded into the sample chamber. The edge jump of Se is identified to be around 1 before compression. High-quality, glitch-free XAFS spectra were obtained under non-hydrostatic pressures up to 53 GPa in the first round of experiment and 63 GPa in the second round of experiment. Pressure was determined by the ruby fluorescence technique. The XANES spectra were simulated using the FDMNES code^42^. The extended x-ray absorption fine structure (EXAFS) spectroscopy was background subtracted, normalized, and Fourier transformed (FT) through standard procedures by ATHENA program.

### First-principles calculations and crystal structure prediction

In order to find a suitable structure to explain the XRD experimental data in the pressure range of 30–65 GPa, we performed fixed-composition structure prediction using the USPEX code at 30, 50, 70 GPa with 4 and 6 formula units of VSe_2_^43–45^. The searching process was terminated when the iteration is more than 20 generations with 50 individual structures per generation.

Structural relaxations and electronic property calculations were carried out via the generalized gradient approximation using the Perdew-Burke-Ernzerhof functional (PBE-GGA), as implemented in the Vienna *ab inito* simulation package (VASP)^46–48^. The projector augmented wave (PAW) method and plane-wave energy cutoff of 700 eV with a dense k-point grid of spacing 2π × 0.03 Å^−1^ in the Monkhorst–Pack scheme were used to sample the Brillouin zone and ensure the structural relaxations with energy and forces converged to less than 10^−7^ eV and 0.01 eV Å^−1^, respectively. Grimme DFT-D3 corrections were applied to take van der Waals interactions between the VSe_2_ layers into consideration. To obtain reasonable pressure-enthalpy curves in Fig. 5, the strongly constrained and appropriately normed (SCAN) meta-generalized gradient approximation (Meta-GGA) was applied to describe the exchange–correlation potential^49^.

The modulated 3D-CDW state (4a × 4a × 3c superstructure) is obtained from Ref.^50^ and is fully optimized to its local minimum on potential energy surface. In order to obtain the 2D structures of various CDW orders in the VSe_2_ monolayer, we first select a suitable lattice vector to build the superlattices with an 18 Å vacuum layer introduced to prevent interlayer interaction. Then, a random displacement of all atom in-plane coordinates in the ranges of 1–3% is imposed on the structure^25^. Finally, full ionic relaxation of the distorted structure is carried out using the parameter (ISIF = 4) in VASP to obtain the final structures.

## Results and discussion

### Pressure-induced 1T-CDW transition in bulk VSe_2_

**Figure 2:**
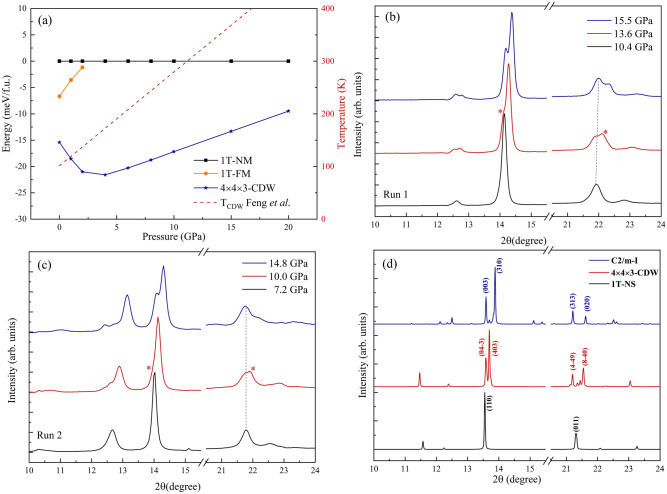
(**a**) Enthalpy curves of ferromagnetic 1*T*-VSe_2_ (*1T*-FM) and the 4a × 4a × 3c CDW superstructure relative to the nonmagnetic VSe_2_ as a function of pressure. Enthalpies are given per formula unit. The pressure-dependent *T*_CDW_ from Ref. 22 is added with Y-axis on the right side of the figure. (**b**,**c**) XRD pattern for the two runs of experiments, the indication of CDW order are marked by red asterisk. (**d**) Calculated XRD pattern for the *C2/m*, CDW, and 1*T*-VSe_2_ structures.

The 1T → CDW transition depends on the relative energy of both structures and the pressure-dependent transition temperature *T*_CDW_. The enthalpy value of the nonmagnetic 1*T*-VSe_2_ (1*T*-NM), ferromagnetic 1*T*-VSe_2_ (1*T*-FM), and 4a × 4a × 3c CDW superstructure are calculated in the pressure range of 0–20 GPa. As shown in Fig. 2a, the CDW order is found to be energetically preferred than FM or NM configuration of 1*T*-VSe_2_, which indicates that it should be the ground state of bulk VSe_2_ at 0 K, in line with experimental observations. In addition, the GGA-PBE calculations display that the CDW order is slightly enhanced in the initial 0–4 GPa and then gradually suppressed until vanishing completely due to a phase transition at 15 GPa. While the FM order in the bulk 1*T*-VSe_2_ is totally suppressed under compression. The pressure-dependent *T*_CDW_ has been clearly established by the electrical transport measured^22^, which is linear enhancement with a constant coefficient. This relationship has been added in Fig. 2a to demonstrating that that the CDW transition approaches room-temperature at about 10 GPa. Since the *C2/m* phase appears at 15 GPa, the room-temperature CDW order can only appear in the narrow pressure range of 10–15 GPa.

We performed two runs of high-pressure XRD experiments for 1*T*-VSe_2_ with special attention to the pressure range of 10–15 GPa. As shown in Fig. 2b, two extra shoulders (labeled by red asterisks) appeared at 13.6 ± 0.4 GPa for the (110) and (011) reflection peaks of the 1*T* phase. It appears that the new peaks are not caused by the broadening of the 1*T* diffraction peaks. Moreover, the profile of the diffraction peak around 22 degree is different from the *C2m*-I phase at 15.5 ± 0.5 GPa. In order to distinguish the three phases, we calculated the theoretical XRD profile of 1*T*, CDW, and *C2/m*-I structures as shown in Fig. 2d. There are a lot of extra satellite peaks appeared in the theoretical XRD profile, which is in fact not observed in our polycrystalline sample due to their weak intensity. However, the theoretical profile based on the fully relaxed 3D-CDW superstructure predicts the splitting of two main peaks, which coincides well with the experimental observations at ~ 14 and ~ 22 degree in Fig. 2b,c. The Rietveld refinements results shown in Figure S1 also support the above hypothesis. Thus, it was considered that the emergence of the shoulder peaks may be caused by the advent of CDW order.

**Figure 3:**
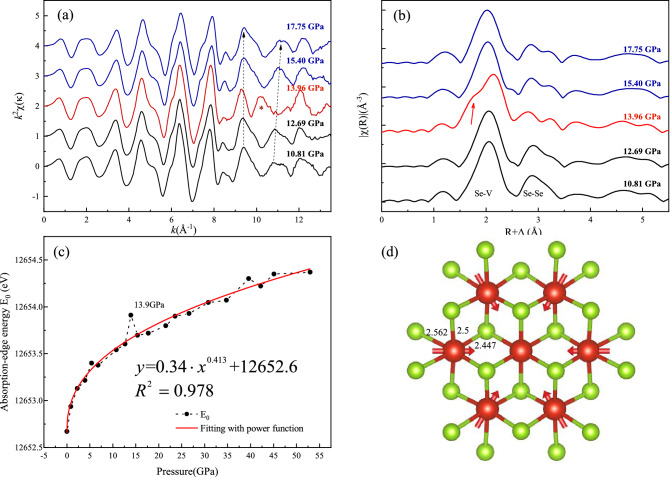
(**a**) The Se *K*-edge EXAFS *k*^2^χ(*k*) oscillation signals from 10.81GPa to 17.75GPa. Abnormal oscillation signals are marked by the red asterisk. (**b**) The corresponding Fourier-transformed magnitude with Se-V and Se-Se bond marked. (**c**) Experimental data (black solid dots) and curve fitting (red line) of the pressure dependence of absorption edge energy in VSe_2_. (**d**) The calculated real space displacement pattern of the 2D-CDW state. The displacement of V atoms across the *1T*-CDW transition is marked by the red arrow, the splitting of Se-V bond length is also shown.

In addition to the lattice information provided by the diffraction pattern, high-pressure XAFS experiments were performed to monitor the evolution of the local atomic structure and the corresponding electronic state during the transition process in VSe_2_^51^. Figure 3a,b shows the pressure dependence of Se *K*-edge EXAFS *k*^2^χ(*k*) oscillation signals^52^ and their Fourier transforms (FTs) for VSe_2_ in the pressure range of 10–17 GPa. The Fourier transforms were characterized by two distinct peaks: the first peak at around 2.0 Å was ascribed to the nearest neighboring Se-V bond, and the other one at around 2.8 Å was associated with the Se-Se bond. By increasing the pressure, the V-S coordination peak displayed small variations in the range of 10.81–12.69 and 15.4–17.5GPa, while the Se-Se FT peak was obviously changed due to the *1T*-*C2/m* phase transition. Surprisingly, the EXAFS signal of 13.96 ± 0.40 GPa is significantly different from others, especially those in the vicinity of *k* = 10 Å^−1^ (marked by a red asterisk in Fig. 3a). The high-*k* oscillation signals correspond to a low *R* peak of the Se-V bond as shown in Fig. 3b. This Se-V shoulder may be associated with the structural distortion that occurred in the 1*T*-CDW transition explained in Fig. 3d, in which the single Se-V bond length is split into multiple bond lengths. This short Se-V bond has not been observed in other pressure ranges and does not belong to the 1*T* or *C2/m*-I phases. Moreover, the pressure-dependent absorption edge energy in Fig. 3c follows a power function relationship. It can be observed that there is an obvious jitter at the 13.96 ± 0.40 GPa for the *E*_*0*_. It deviates from the fitted curve (red dotted line), implying a drastic change of the final state energy, i.e., lowest unoccupied state, in the dipole transition of 1 s → 4p in Se element. It probably origins from the change in the electron density of the conduction band in the process of CDW formation, which provided a spectroscopic signature for the metallic 1*T* phase to the semiconductor-like CDW state.

### New high-pressure phase of bulk VSe_2_

**Figure 4:**
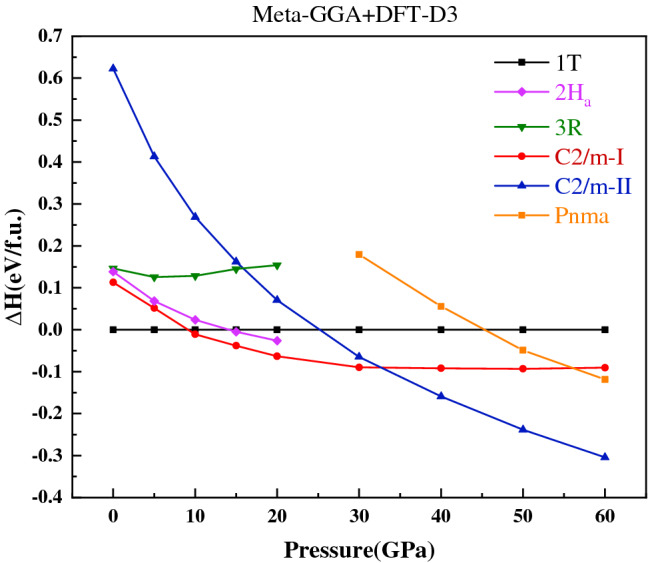
The pressure-enthalpy curve of 1*T*, 2*H*_*a*_, 3*R*, *C2/m*-I, *C2/m*-II, and *Pnma* structures in bulk VSe_2_. The 1*T*-FM state was used as a reference ground state. The calculation in this figure is based on the Meta-GGA exchange–correlation potential with van der Waals (vdW) interactions considered in the DFT-D3 approach.

Combining crystal structure prediction and in situ X-ray measurements, we identify a new monoclinic VSe_2_ structure (labeled as *C2/m*-II) in the pressure range of 35–65 GPa. It is generated by the USPEX code and has a similar structure with the *C2/m*-I phase. The main difference between the two structures is the Wyckoff position of the V atom and the *β* angle of the lattice. In addition, the coordination number (CN) of the V atom in *C2/m*-II is 8, while the CN is 6 in 1*T* and *C2/m*-I phase. Figure 4 shows the pressure-enthalpy curve of various structures in the whole pressure range of 0–60 GPa. The *C2/m*-II structure was found to have a lower energy than all previously proposed structures in the pressure range of 40–60 GPa. Meanwhile, the phonon dispersion calculations with no imaginary frequencies further confirmed the dynamical stability^53^ of the *C2/m*-II phase under 50 and 70 GPa. The pressure-enthalpy curve reveals a complete phase transition pathway of 1*T* → *C2/m-*I → *C2/m-*II and is compatible with the XRD and XAFS experiments. The theoretical phase transition pressure of the 1*T* → *C2/m*-I is 10 GPa at low temperature, which is smaller than the actual value of 15 GPa identified by the room-temperature XRD experiments. The 3*R* phase of VSe_2_, which is recommended to be the high-pressure phase in the recent report^54^, was found to be less stable than the 1*T* and *C2/m*-I structures in the 0–20 GPa. In addition, we also predicted a non-layered structure of VSe_2_ with *Pnma* symmetry, which was added in Fig. 4 for comparison. The detailed crystal structures and their cell parameters are reported in Table S1 in supplementary materials.

**Figure 5:**
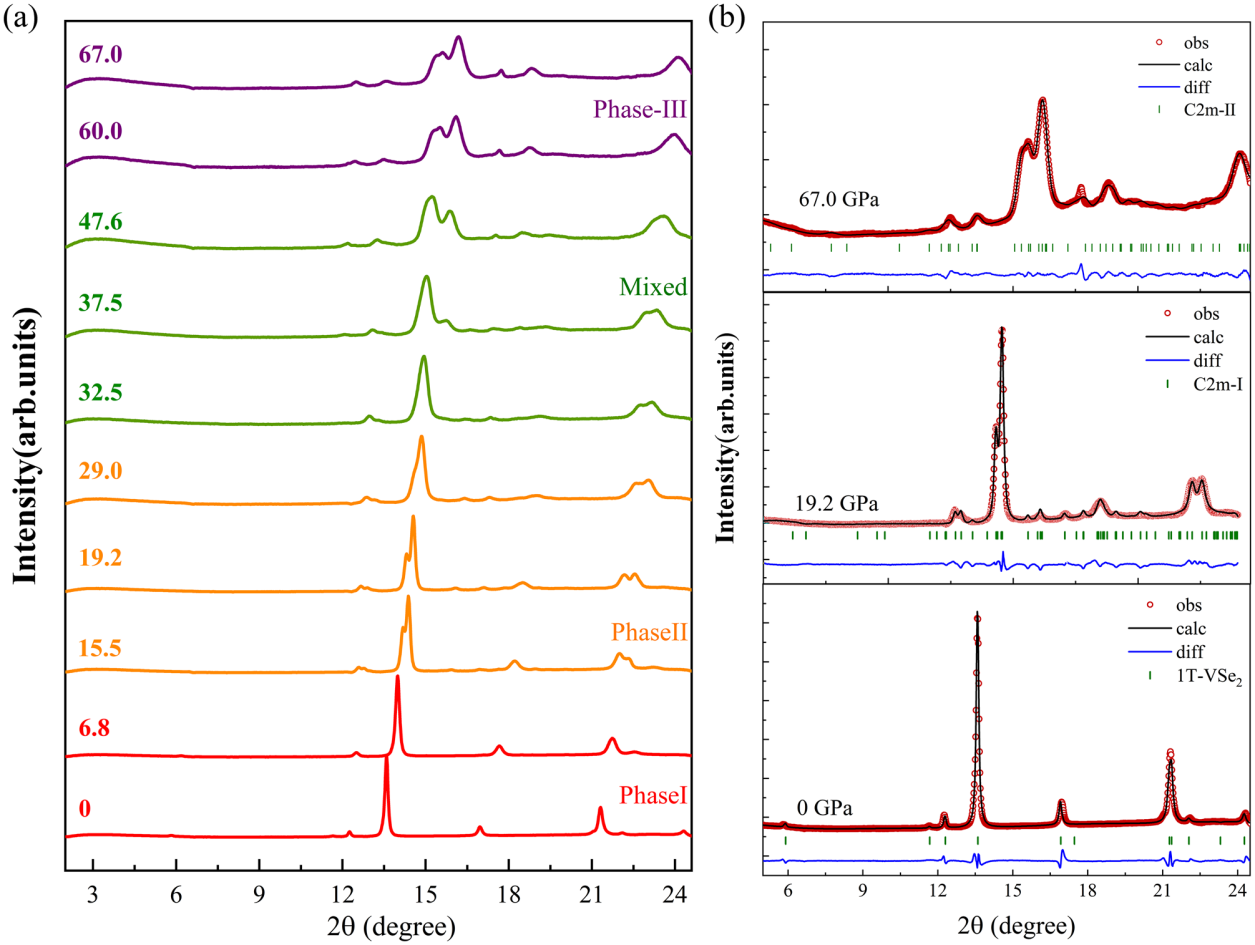
(**a**) selected data of high-pressure XRD experiments up to 67.0 GPa. (**b**) Experimental (red circles) and Rietveld-refined XRD pattern (black line) for bulk VSe_2_ at 0, 19.2, and 67.0 GPa, respectively. The solid blue lines at the bottom are the residual intensities and the vertical bars indicate the peak positions. The refining parameters are *R*_*wp*_ = 3.2%, *χ*^*2*^ = 0.45 for 1T phase, *R*_*wp*_ = 2.6%, *χ*^*2*^ = 0.30 for *C2/m*-I, and *R*_*wp*_ = 1.5%, *χ*^*2*^ = 0.11 for *C2/m*-II phase, respectively.

**Figure 6:**
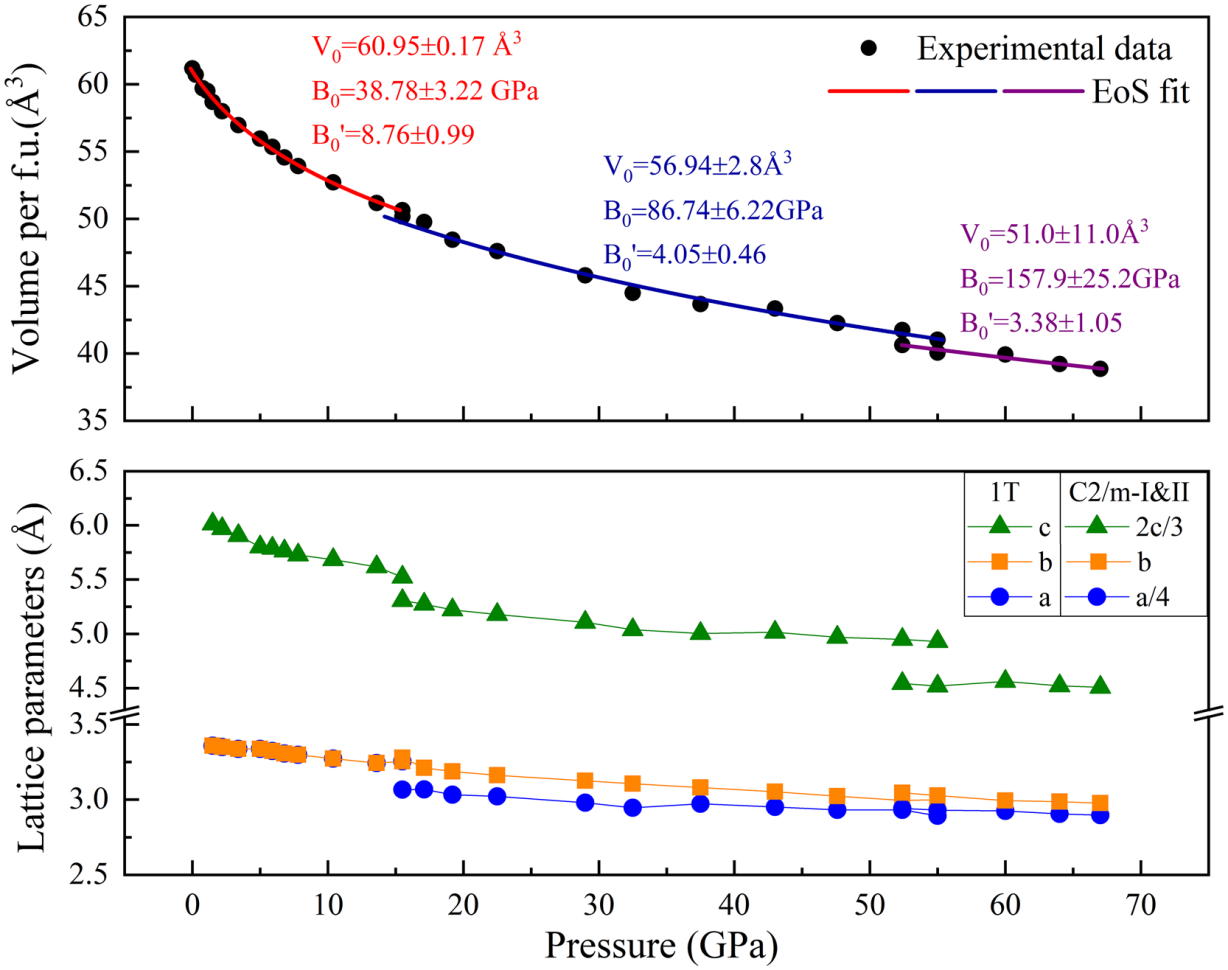
Pressure dependence of the unit cell volume, lattice constants a, b, and c for the VSe_2_ in the pressure range of 0–67 GPa.

Figure 5 shows the selected data of high-pressure XRD experiments and the Rietveld refinements results based on the 1*T*, *C2m*-I, and *C2/m*-II structures of VSe_2_. The detailed refined structural parameters are reported in Table S2. The main difference from the previous experiments is the appearance of the new peak at ~ 15 degree, which first appeared at ~ 37.5 ± 1.1 GPa, gradually enhanced until ~ 60 ± 2 GPa, and finally became stable up to ~ 67 ± 2 GPa. The experimental observation can be well explained by the 8-coordination monoclinic *C2/m*-II structure. For the pressure range of 32.5–47.6 GPa, VSe_2_ is probably in a transition state or a mixed phase of the *C2m*-I and *C2m*-II. The pressure dependence of the unit cell volume and lattice constants are reported in Fig. 6, which is in good agreement with the theoretical results in Figure S2. The current theoretical and experimental results show that there is volume collapse during the phase transition of *C2m*-I to *C2m*-II.

**Figure 7:**
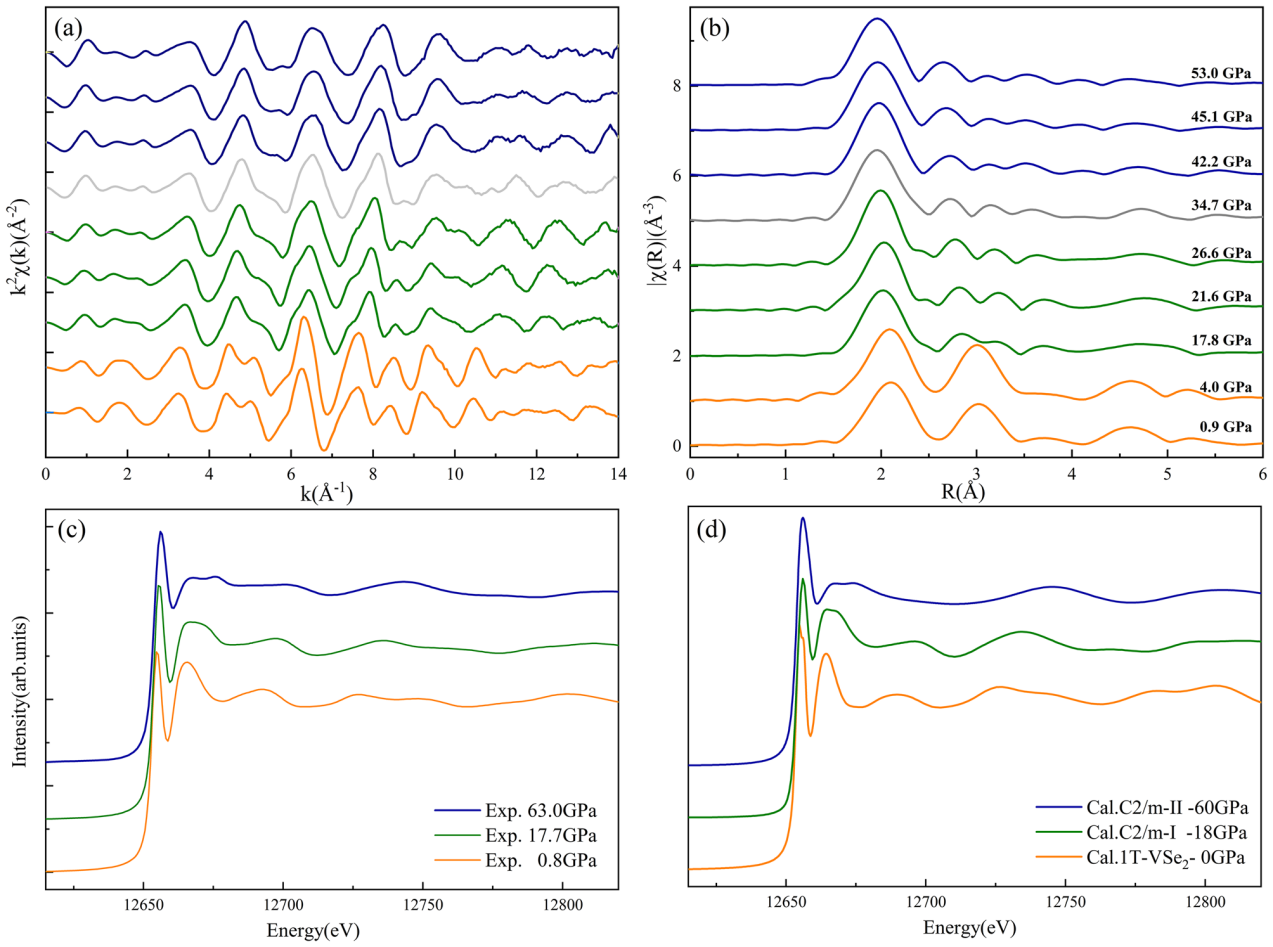
(**a**) The Se *K*-edge EXAFS *k*^2^χ(*k*) oscillation signals of bulk VSe_2_ collected at different pressures in the range of 0–53.0 GPa. (**b**) The corresponding Fourier-transformed magnitude for bulk VSe_2_. (**c**) Normalized Se K-edge XANES experimental spectra of bulk VSe_2_ under the pressure of 0.8, 17.7, and 63.0 GPa. (**d**) The simulated spectrum of VSe_2_ using FDMNES code based on the 1*T*, *C2/m*-I, and *C2/m*-II structures.

Figure 7 shows the evolution of the Se *K*-edge XAFS spectra of bulk VSe_2_. Two runs of experiments were performed with no significant difference found between them, except that the maximum pressure was ~ 53 ± 2 GPa for the first round and ~ 63 ± 2 GPa for the second round. The measured spectra at various pressures can be divided into three categories based on the XANES features and EXAFS signals. The main characteristic of the first phase transition is the splitting of the peak at ~ 3 Å under ~ 17.8 ± 0.5 GPa in Fig. 7b, which indicates the rearrangement of Se atoms in the second shell. The key characteristic of the second phase transition is the appearance of a new XANES features at ~ 12,676 eV in Fig. 7c. The change of the XAENS features can be well simulated by the phase transition pathway of 1*T* → *C2/m-*I → *C2/m-*II shown in Fig. 7d, indicating that it is reasonable to assign the new phase of VSe_2_ in the pressure range of 35–65 GPa to be the *C2/m*-II structure. During the second phase transition, the coordination number (CN) of V atoms increased from 6 to 8. The EXAFS fitting shown in Figure S3 clearly supports the phase transition sequence of 1*T* → *C2/m-*I → *C2/m-*II. The derived structural parameters are reported in Table S3. The schematic diagram of the whole transition sequence in VSe_2_ and the changes in the structure of the vanadium trimers^22^ are shown in Figure S4.

## Conclusions

In summary, from first-principles calculations the CDW order is found to be energetically preferred than FM or NM configuration of 1*T*-VSe_2_, which indicates that it should be the ground state of bulk VSe_2_. Based on the high-pressure XRD and XAFS experiments, two experimental anomalies were detected and attributed to the pressure driven 1*T* to CDW transition in bulk VSe_2_. In addition, an eightfold *C*2/m structure was theoretically predicted and experimentally identified to be a new phase of bulk VSe_2_ in the pressure range of 35–65 GPa. These findings not only help to determine the ground-state crystal structure of VSe_2_ but also help to understand the high-pressure behavior of the charge-ordered state.

## Supplementary Information

Supplementary Information.
