# Supplementary material for: Observation of pressure induced charge density wave order and eightfold structure in bulk VSe2
Source: Sci Rep. 2021 Sep 13;11:18157. doi: 10.1038/s41598-021-97630-8 (PMC8437963; doi:10.1038/s41598-021-97630-8)
Supplement: Supplementary file 1 — Supplementary Information. [file 41598_2021_97630_MOESM1_ESM.docx]

**Supplementary Materials**

**Observation of pressure induced charge density wave order and 8-fold structure in bulk VSe_2_**

Zhiying Guo^1^, Xingyu Hao^1,2^, Juncai Dong^1^, Haijing Li^1,2^, Jiangwen Liao^1,2^ and Dongliang Chen^1,^ *

*^1^ Beijing Synchrotron Radiation Facility, Institute of High Energy Physics, Chinese Academy of Sciences, Beijing 100049, China*

*^2^ University of Chinese Academy of Sciences, Beijing 100042, China*





Figure S1 Rietveld refinements of the XRD patterns collected at 10.4 GPa, 13.6GPa and 15.6 GPa of run 1. The solid blue lines at the bottom are the residual intensities and the vertical bars indicate the peak positions. The refining parameters of weighted R-factor and chi-squared values are reported in the figure.

Table S1. The predicted crystal structures and their cell parameters after fully structural relaxation in the framework of Meta-GGA + DFT-D3.

| Phase | Space Group | Lattice Parameter  (Å) | Atom | Wyckoff  Position | Atomic Coordinates | | | Bond Length |
| --- | --- | --- | --- | --- | --- | --- | --- | --- |
|  |  |  |  |  | X Y Z | | |  |
| **1T**  **@0GPa** | $\boldsymbol{P}\bar{\mathbf{3}}\boldsymbol{m}\mathbf{1}$ | **a= 3.3916**  **c=** **6.0306** | **V**  **Se** | **1a**  **2d** | **0**  **0.3333** | **0**  **0.6667** | **0**  **0.25268** | **R_(V-Se)_ = 2.481** |
|  |  | **γ=120°** |  |  |  |  |  |  |
| **C2/m-I**  **@19GPa** | ***C2/m*** | **a= 11.9794**  **b=** **3.1240**  **c=** **7.7452**  **β=106.84°** | **V1**  **V2**  **Se1**  **Se2**  **Se3** | **2a**  **4i**  **4i**  **4i**  **4i** | **0**  **0.6403**  **0.9993**  **0.3021**  **0.6537** | **0**  **0**  **0**  **0**  **0** | **0**  **0.2943**  **0.3147**  **0.3733**  **0.0054** | **R_(V1-Se1)_ = 2.440**  **R_(V1-Se3)_ = 2.406**  **R_(V2-Se1)_ = 2.339**  **R_(V2-Se2)_ = 2.425**  **R_(V2-Se3)_ = 2.289** |
| **C2m-II**  **@67GPa** | ***C2/m*** | **a= 11.4158**  **b= 2.8949**  **c= 6.6454**  **β=** **92.11°** | **V1**  **V2**  **Se1**  **Se2**  **Se3** | **2a**  **4i**  **4i**  **4i**  **4i** | **0**  **0.1858**  **0.3426**  **0.5256**  **0.1714** | **0**  **0**  **0**  **0**  **0** | **0**  **0.2539**  **0.0278**  **0.3035**  **0.6066** | **R_(V1-Se1)_ = 2.320**  **R_(V1-Se2)_ = 2.491**  **R_(V2-Se1)_ = 2.379**  **R_(V2-Se2)_ = 2.366**  **R_(V2-Se3)_ = 2.345** |
| Pnma  @50GPa | *Pnma* | a= 6.6782  b=2.8888  c=7.9105  α=β=γ=90° | V  Se1  Se2 | 4c  4c  4c | 0.1764  0.3429  0.0318 | 0.25  0.25  0.25 | 0.1751  0.4400  0.6506 | R_(V-Se1)_ = 2.358  R_(V-Se2)_ = 2.433 |

Table S2. The refined structural parameters of 1*T*, *C2/m*-I and *C2/m*-II phase of bulk VSe_2_.

| Phase | Space Group and | Lattice Parameter  (Å) | Atom | R-factor and chi-squared values | Atomic Coordinates | | |
| --- | --- | --- | --- | --- | --- | --- | --- |
|  |  |  |  |  | X Y Z | | |
| **1T**  **@0GPa** | $\boldsymbol{P}\bar{\mathbf{3}}\boldsymbol{m}\mathbf{1}$ | **a= 3.3831**  **c=** **6.1763**  **γ= 120°** | **V**  **Se** | **R_wp_=3.2%**  **χ^2^ =0.45** | **0**  **0.3333** | **0**  **0.6667** | **0**  **0.2500** |
| **C2/m-I**  **@19GPa** | ***C2/m*** | **a= 12.055**  **b= 3.1756**  **c= 7.7801**  **β=105.78°** | **V1**  **V2**  **Se1**  **Se2**  **Se3** | **R_wp_=2.6%**  **χ^2^ =0.30** | **0**  **0.6666**  **0.0513**  **0.3318**  **0.6308** | **0**  **0**  **0**  **0**  **0** | **0**  **0.3145**  **0.3377**  **0.3319**  **-0.0070** |
| C2m-II  @67GPa | *C2/m* | a= 11.916  b=2.9915  c=6.9270  β=94.56° | V1  V2  Se1  Se2  Se3 | R_wp_=1.5%  χ^2^ =0.11 | 0  0.1870  0.3453  0.5333  0.1747 | 0  0  0  0  0 | 0  0.2522  0.0301  0.3102  0.6095 |





Figure S2. The calculated pressure dependence of the unit cell volume, lattices parameters, and β angle for the *1T* and *C2/m*-I and *C2/m*-II phases of bulk VSe_2_ in the range of 0-70 GPa. By fitting the pressure-volume data with third-order Birch-Murnaghan equation of state, the value of theoretical equilibrium volume, bulk modulus B_0_, and pressure derivative of the bulk modulus B_0_′ of the 1T phase VSe_2_ are V_0_=60.09±0.1 Å^3^, B_0_=32.31±1.24 GPa, and B_0_'=7.13±0.33.

Figure S3. The experimental EXAFS spectrum (black circles) of bulk VSe_2_ collected at 0.87, 26.6 and 53.0 GPa and the best-fit results (red line) in the range of 1.6-3.5Å based on the 1T, *C2/m*-I and *C2/m*-II structure.

Table S3. The structural parameters (distance R, coordination number N and Debye-Waller factor σ^2^) for the different Se-V and Se–Se pairs for VSe_2_ samples for the three phases during the compression process.

| Pressure GPa | Bond | R(Å) | N | σ^2^(10^-3^ Å) |
| --- | --- | --- | --- | --- |
| 0.87 | Se-V | 2.48±0.01 | 3 | 8.1±0.6 |
|  | Se-Se | 3.19±0.04 | 3 | 8.4±3.3 |
|  | Se-Se | 3.33±0.02 | 6 | 7.1±1.5 |
| 26.6 | Se-V | 2.32±0.01 | 1 | 4.3±0.8 |
|  | Se-V | 2.42±0.01 | 2 | 4.3±0.8 |
| 53.0 | Se-V | 2.37±0.02 | 3 | 4.4±1.9 |
|  | Se-V | 2.40±0.02 | 0.333 | 4.4±1.9 |
|  | Se-V | 2.51±0.02 | 0.667 | 4.4±1.9 |


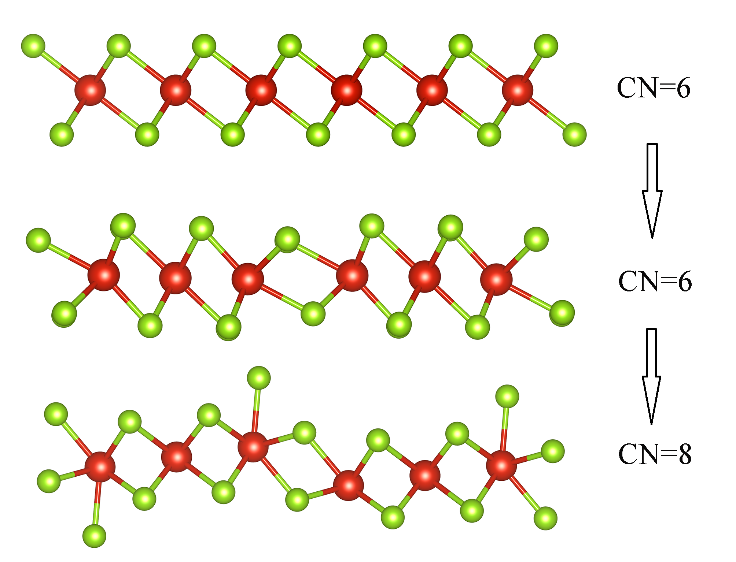


Figure S4. Changes in the structure of the vanadium trimers and the pressure-induced increase of vanadium coordination number (CN) from six to eight.
